# The Effects of *Banha-sasim-tang* on Dyspeptic Symptoms and Gastric Motility in Cases of Functional Dyspepsia: A Randomized, Double-Blind, Placebo-Controlled, and Two-Center Trial

**DOI:** 10.1155/2013/265035

**Published:** 2013-06-03

**Authors:** Jae-Woo Park, Seok-Jae Ko, Gajin Han, Inkwon Yeo, Bongha Ryu, Jinsung Kim

**Affiliations:** ^1^College of Korean Medicine, Kyung Hee University, 26 Kyungheedae-ro, Dongdaemun-gu, Seoul 130-701, Republic of Korea; ^2^College of Sookmyung Women's University, Cheongpa-dong 2ga, Yongsan-gu, Seoul 140-132, Republic of Korea

## Abstract

*Introduction*. Functional dyspepsia (FD) is highly prevalent, and no standard treatments exist for this condition. Herbal prescriptions are widely used to treat FD. In traditional Korean medicine, *Banha-sasim-tang* (BST) is a famous herbal prescription for dyspepsia. This study aimed to evaluate the efficacy of BST and to examine the relationship between gastric slow waves and dyspeptic symptoms. *Materials and Methods*. In total, 100 FD patients were recruited; BST or placebo was administered for 6 weeks. The gastrointestinal symptom scale, FD-related quality of life scale, and frequency or power variables regarding gastric slow waves were measured at 0, 6, and 14 weeks. *Results*. There were no significant differences in the overall dyspeptic symptoms or quality of life between the BST and placebo groups. However, early satiety was significantly improved in the BST group (*P* = 0.009, at 6 weeks by intention-to-treat analysis). Abnormal gastric dysrhythmias and power ratios were also significantly improved by BST. *Conclusion*. BST had no significant effects on FD. However, early satiety appeared to improve after BST administration. Electrogastrography may be a useful technique for assessing changes in gastric motility dysfunction after interventions for FD. Further investigation focused on specific symptoms or subtypes of FD is required.

## 1. Introduction

Functional dyspepsia (FD) is chronic or recurrent dyspepsia without evidence of organic disease [1]. In western countries, the prevalence of FD is 11.5–14.7% in the general population [2]. In South Korea, an epidemiologic survey showed that 25% of the population was afflicted by FD [3].

At present, the causes of FD are believed to be multifactorial, and pathogenesis-based treatments, including administration of acid secretion inhibitors and prokinetics and eradication of *Helicobacter pylori*, have been used without success [4–6]. Therefore, many FD patients seek alternative and more effective treatments such as herbal prescriptions, nutritional supplements, acupuncture, or moxibustion [1, 7–9].

*Banha-sasim-tang* (BST; *Hange-shashin-to* in Kampo medicine; *Ban xia xie xin tang* in traditional Chinese medicine) is one of the most famous herbal prescriptions in the old herbal prescription literature, where it is referred to as “*Shan-han-za-bing-lin* (*傷寒雜病論*)” [10]. In traditional Korean medicine, BST, which consists of 7 herbs, has been used to treat “gastric stuffiness” [10], a condition similar to dyspepsia. Recently, some studies have reported that BST has anti-inflammatory effects on the gastric mucosa and affects gastric function by influencing gut hormones or plasma peptides [11–13]. A recent clinical study investigating modified BST, conducted in China, demonstrated beneficial effects on only a subgroup of FD patients [14]. BST can be easily obtained without a prescription in Korea. Despite many previous experimental and clinical research studies, including a randomized controlled trial (RCT) in China [14], more reliable evidence about BST as a therapeutic alternative for FD are needed. However, few relevant RCTs have examined the effects of BST on FD or its mechanisms. The aim of the present study was to investigate the effect of BST on dyspeptic symptoms and the quality of life in FD patients. To assess the changes in gastric motility dysfunction caused by BST, as a mechanical study, cutaneous multichannel electrogastrography (EGG), which detects gastric myoelectric activity (GMA), was used. The relationship between EGG frequency variables and FD symptoms was analyzed before and after oral administration of BST.

## 2. Materials and Methods

### 2.1. Study Design

This study was conducted as a randomized, placebo-controlled, double-blind, and 2-center trial at the Oriental Hospital, Kyung Hee University Medical Center, and at the Kyung Hee University Hospital at Gangdong in Seoul, Korea. The protocol of the trial was approved by the ethics review boards of both hospitals. The permission numbers were KOMC IRB 2009-05 for the Oriental Hospital and KHNMC-OH-IRB 2009-001 for the Kyung Hee University Hospital. Written informed consent was obtained from each participant before enrollment.

### 2.2. Study Subjects

Patients aged 19–75 and meeting the Rome III criteria for FD [15] were recruited from the clinics at both hospitals and from responders to local advertisements. In this trial, FD patients were classified into 1 of 3 subtypes: PDS, EPS, or mixed type: (1) meal-induced postprandial distress syndrome (PDS), characterized by postprandial fullness and early satiety; (2) epigastric pain syndrome (EPS), characterized by epigastric pain and burning; (3) PDS mixed with EPS [15]. The severity of dyspepsia was assessed using a validated gastrointestinal symptom (GIS) scale, which consists of 10 dyspeptic symptom subscales and is used to measure the severity of individual dyspeptic symptoms [16]. The existence of “moderate” severity on at least 3 subscales of the GIS resulted in the patient meeting the study's inclusion criteria [1]. However, patients with medical histories of peptic ulcers, reflux diseases, previous abdominal surgeries, mental diseases, such as major depression, predominant irritable bowel syndrome, severe organ diseases, or continuous administration of analgesic agents were excluded as were lactating or pregnant women. In addition, patients who were using antibiotics, proton-pump inhibitors, bismuth salts, prokinetic agents, and herbal prescriptions were excluded, as previously described [17].

### 2.3. Randomization and Blinding

Randomization was performed by an independent clinical research coordinator (CRC). The randomization document with the subject's basic information was transmitted by facsimile to the independent statistician (IKY) without a confirmed randomization number. The statistician determined the randomization number based upon the allocation sequence generated in advance by a random number program. Subsequently, the statistician returned the randomization document with the confirmed number to the CRC. The random allocation ratio for the 2 sites was 1 : 1. The randomization process was assured by the authorized contract research organization (CRO; Marinet, Seoul, Korea), as previously described [17].

During the study, the investigators did not contact the CRC, the clinical pharmacist, or the statistician. In particular, the CRC was separated from all researchers to ensure that the researchers did not have any influence on enrollment or randomization. The statistician received the randomization document with the number and returned it to CRC in order. Thus, the statistician's contact with other investigators was avoided. This blinding procedure was also verified by the authorized CRO.

Thus far, no standard therapy has been established for FD; therefore, the placebo used in this study did not have any active components. As experimental agents, Bansasin granules (Hanpoong Pharm & Food, Jeonju, Korea) in the current study are not artificial chemical therapeutics; a relevant placebo with an identical appearance, color, and flavor was required for effective blinding. After several attempts, a placebo that could not be distinguished from the real Bansasin granule by 6 healthy persons was successfully produced. Placebo was made of starch, lactose, brown caramel food coloring (BF2481, SaeRom BNF Co., Korea), and flavor (BF24781, SaeRom BNF Co., Korea) similar to color, flavor, and scent with Bansasin granule. Samples of the placebo were also kept by Hanpoong Pharm & Food. Additionally, at the end of the study, all subjects were asked whether the experimental agents that they had been given were real or placebo in order to evaluate the success of blinding.

### 2.4. Interventions

The intervention of the current study, BST (1/3 pack of herbal medicine (*貼*): pinelliae tuber (the rhizome of *Pinellia ternata* (Thunb.) Breit., family Araceae), 1.67 g; scutellariae radix (the root of *Scutellaria baicalensis *Georgi, family Labiatae), 1.00 g; ginseng radix (the root of *Panax ginseng* C.A. Meyer, family Araliaceae), 1.00 g; glycyrrhizae radix (the root of *Glycyrrhiza uralensis *Fisch., family Leguminosae), 1.00 g; zizyphi fructus (the fruit of *Zizyphus jujuba* Mill. var. inermis Rehder, family Rhamnaceae), 1.00 g; zingiberis rhizoma (the rhizome of *Zingiberis officinale* Roscoe, family Zingiberaceae), 0.83 g; coptidis rhizoma (the rhizome of *Coptis chinensis *Franch., family Ranunculaceae), 0.33 g) was extracted (0.91 g) with boiled water and mixed with starch (1.57 g) and lactose (0.52 g) then given the Bansasin granule (3 g). The manufacture was processed according to Korean Good Manufacturing Practice and permitted and regulated by the Korean Food & Drug Administration. The standard chemical components in the Bansasin granule (3 g) are berberin (11.6 mg), glycyrrhizin acid (25.0 mg), and baicalin (100.0 mg). Sample specimens were kept at the laboratory of Hanpoong Pharm & Food. Generally, 3 g of Bansasin granules 3 times per day is the recommended adult dosage for dyspeptic symptoms, including nausea, vomiting, diarrhea, abdominal pain, or anorexia [17].

This clinical trial consisted of a 6-week oral administration of BST or placebo (3 g, 3 times/day) and a 2-month follow-up period. Before randomization, all participants underwent a 7-day washout phase for elimination of any traces of previous medications. During the administration phase, subjects were prohibited from taking any kind of dyspepsia-relieving agents. However, conventional treatments for dyspepsia were permitted if the dyspeptic symptoms were severe and could not be tolerated. During the follow-up period, any other type of treatment provided to the subjects was reported and documented.

### 2.5. Outcomes

Dyspepsia severity and quality of life were measured at baseline; at 2, 4, and 6 weeks after randomization; and at 1 and 2 months after completion of the BST or placebo administration.

The GIS scale was the primary outcome variable in this study [16], and the change in the total score of the GIS scale or significant changes in the GIS subscale scores at 6 weeks were considered as efficacy parameters. The GIS scale is composed of the following 10 dyspeptic symptoms: epigastric pain/upper abdominal pain, abdominal cramps, fullness, early satiety, loss of appetite, malaise, nausea, vomiting, retrosternal discomfort, and acidic regurgitation/heartburn. The severity of each subscale symptom was assessed using a 5-point Likert scale: none, 0; slight, 1; moderate, 2; severe, 3; very severe, 4. The total sum of the 10 GIS subscale scores presents patient's overall severity of dyspeptic symptoms. If the total sum of the GIS scale is higher, then the dyspeptic symptoms are more severe. All subjects assessed their own symptom severity by themselves. The GIS scale is very simple and easy for subjects to understand and to fill in. 

As a secondary outcome, a visual analog scale (VAS) was used to determine the patient's overall judgment about dyspepsia (ranging from 0, no discomfort, to 100, the most intense discomfort). 

For assessing the quality of life, the functional dyspepsia-related quality of life (FD-QoL) questionnaire, as a secondary outcome, was used for all subjects. The questionnaire consisted of 4 categories: diet (5 items), daily activity (4 items), emotion (6 items), and social functioning (6 items) [18]. The severity assessment for each item was the same as that for the GIS subscale. The FD-QoL questionnaire used in this trial was previously validated for use with Korean FD patients [18]. The total sum of the FD-QoL item scores presents patient's overall quality of life related dyspepsia. If the total sum of the FD-Qol item scores is lower, then the quality of life related to dyspepsia is better. All subjects assessed their own state by themselves.

### 2.6. GMA Measurement

In the present study, surface multichannel EGG (Polygraf ID, Medtronic A/S, Copenhagen, Denmark) was used to measure GMA in each subject at baseline and at 6 weeks of BST or placebo administration. As previously described [19], EGG measurements were conducted in the following sequence. First, the epigastric skin at the electrode attachment site was shaved and abraded with a sandy skin preparation jelly to reduce impedance. Then, 3 active surface electrodes were positioned at the following sites: the corpus of the stomach (channel 1), the proximal antrum (channel 2), and the distal antrum and pylorus regions (channel 3). A ground electrode and a reference electrode were also appropriately placed. EGG measurements were obtained in a quiet room after the subjects had fasted over night for ≥8 hours. To avoid motion artifacts, subjects were asked not to talk and to remain as still as possible during the EGG assessments. Each subject underwent a fasting (preprandial) EGG measurement for 20 minutes in the supine position and then ate the standard solid test meal (500 Kcal, 2 scrambled medium eggs, and 2 pieces of toasted bread with 500 mL of water). A postprandial EGG measurement was then performed for 40 minutes. The dominant EGG frequency, the percentage or percentage distribution of normal or abnormal gastric slow waves (tachygastria, bradygastria, and dysrhythmia), and the postprandial-to-preprandial power ratio which is defined as the ratio of the postprandial to fasting power of the dominant frequency (DF) were assessed.

### 2.7. Sample Size Calculation

To determine the efficacy of BST on FD, the superiority of BST over placebo had to be verified. Although there were no relevant previous studies using BST for calculating sample size, a previous herbal prescription trial for FD treatment had assessed efficacy using the GIS scale and a 2-sided test, yielding a 5% significance level [20]. The formula for estimating the sample size was as follows:

(1) $$\begin{array}{c} n_{t}=n_{c}=\frac{\left\{{\left(Z_{\alpha /2}+Z_{\beta }\right)}^{2}\sigma^{2}\left(\lambda +1\right)/\lambda \right\}}{{\left(\mu_{c}-\mu_{t}\right)}^{2}}. \end{array}$$

From the previous study, 3.5 points of GIS scale (*μ*_*c*_ − *μ*_*t*_ = Δ) improved after herbal treatments compared with placebo, and a mean standard deviation (SD = *σ*) was 5.37 [21]. In our study, the ratio (*λ*) of BST to placebo group was 1 : 1. With an 80% power (1 − *β*) and 5% significance level (*α*), assuming Δ = 3.5 and *σ* = 5.37, a sample size of *n*_*t*_ = *n*_*c*_ = 37 subjects per group was needed (*n*_*t*_, number of patients in the BST group; *n*_*c*_, number of patients in the placebo group). Considering an expected dropout rate of 25%, 100 patients were required, as previously described [17].

### 2.8. Statistical Analysis

All analyses in this study were based on the intention-to-treat principle. Quantitative- and frequency-related variables were presented as means ± SD and number (%), respectively. The baseline characteristics of both groups were compared by the chi-square test or an independent *t*-test. As primary outcomes, the changes in the GIS total score or subscale scores from the beginning (day 0) to the end (6 weeks) of the study period were compared using an independent *t*-test. In addition, the VAS results for overall dyspeptic symptoms and the total FD-QoL scores were also compared in the same manner as the GIS scores. Frequency parameters in the EGG measurement, such as the dominant frequency, percentages of normal or abnormal gastric slow waves, and power ratio variables, were compared before and after treatment using the chi-square test or Fisher's exact test. Correlations between changes in the GIS scale results and the EGG parameters were analyzed by Pearson's correlation coefficients. Statistical analyses were conducted in a blinded manner by an independent statistician using SPSS 16.0 (SPSS, Chicago, IL, USA).

## 3. Results

### 3.1. Study Participants

**Figure 1:**
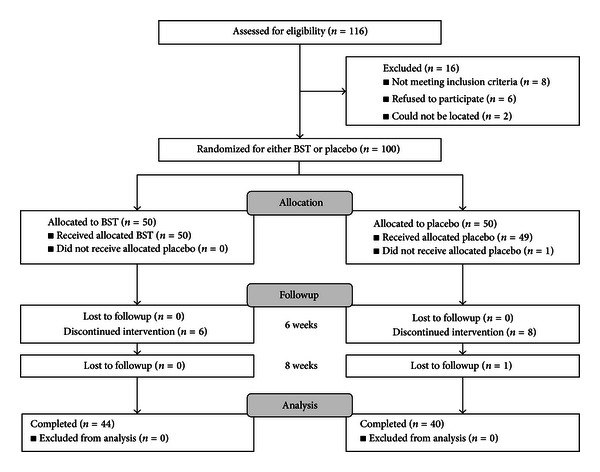
Flow chart of the trial.

Of the 116 eligible subjects, 100 patients were included and randomly allocated to the BST or placebo groups in a 1 : 1 ratio between May 2009 and January 2011. Sixteen subjects withdrew during the study because of lack of efficacy or failure to followup. A flow chart of the trial is presented in Figure 1.

### 3.2. Baseline Characteristics

The general characteristics of the subjects are presented in Table 1. No significant differences were observed in any of the parameters between the 2 groups, except for body weight. However, the characteristics of dyspepsia in the BST group tended to be more continuous than those in the placebo group.

### 3.3. Safety and Adverse Events

Before randomization and after completing administration of BST or placebo, we assessed complete blood cell counts; levels of aspartate aminotransferase/alanine aminotransferase, gamma-glutamyl transpeptidase, blood urea nitrogen, and creatinine; erythrocyte sedimentation rates; electrocardiograms to ensure the subjects' safety. Throughout the trial, all adverse events were also monitored by the subjects' reports or the case report form documentation.

There were no safety issues in this trial. Eight adverse events, including epigastric pain or discomfort (*n* = 3), insomnia (*n* = 2), dry mouth (*n* = 2), and itching (*n* = 1), were reported by 4 subjects in the BST group. Thirteen adverse events, including abdominal pain or discomfort (*n* = 2), insomnia (*n* = 2), constipation (*n* = 2), itching (*n* = 3), dizziness (*n* = 1), and muscle pain (*n* = 3), were reported by 9 subjects in the placebo group. However, there were no serious adverse events during the study period.

### 3.4. Outcome Variables

#### 3.4.1. GIS Scores and Individual Dyspeptic Symptoms

**Figure 2:**
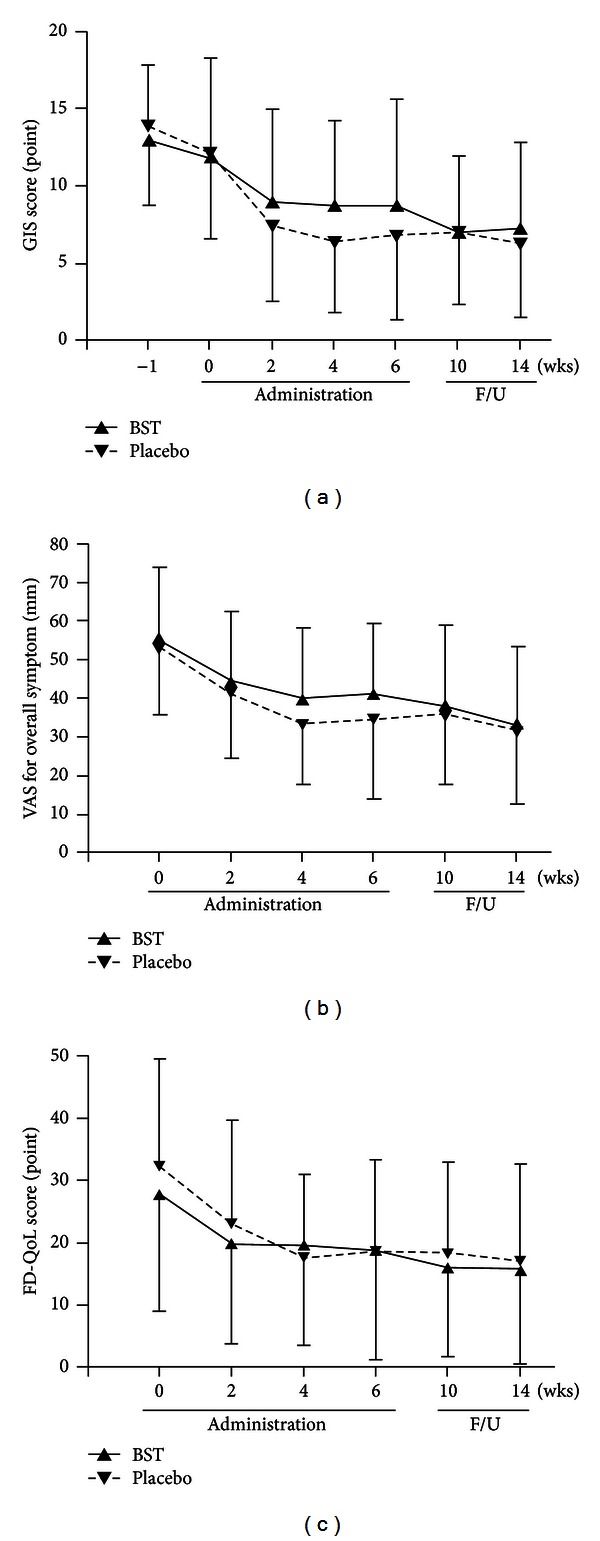
Changes in main outcomes of the trial. (a) Changes in the total scores of gastrointestinal symptom (GIS) scale between BST and placebo groups during the whole trial. (b) Changes in visual analogue scale (VAS) for overall symptoms between BST and placebo groups during the whole trial. (c) Changes in the total scores of functional dyspepsia-related quality of life (FD-QoL) between BST and placebo groups during the whole trial. BST: *Banha-sasim-tang*. F/U: follow-up period.

After 6 weeks of treatment, the total GIS scores for both groups had improved markedly (13.06 ± 4.82 at 0 week to 8.77 ± 6.87 at 6 weeks in BST group versus 13.94 ± 5.12 at 0 week to 6.83 ± 5.42 at 6 weeks in the placebo group); no significant difference was observed between the 2 groups (Figure 2). However, at 6 weeks, the early satiety subscale score was significantly improved in the BST group compared with the placebo group (*P* = 0.009, by intention-to-treat analysis; Table 2). However, there were no significant differences among other subscale scores at 6 weeks between the 2 groups (Table 2). During the follow-up period, the improved GIS scores were retained. However, there were no significant differences in the GIS scores or the individual dyspeptic symptoms between the groups.

#### 3.4.2. VAS for Overall Symptoms

After 6 weeks of treatment, the VAS for overall symptoms in both groups had improved (55.36 ± 18.63 at 0 week to 41.32 ± 18.21 at 6 weeks in BST group versus 53.08 ± 17.29 at 0 week to 34.54 ± 20.62 at 6 weeks in the placebo group); no significant difference was observed between the 2 groups (*P* = 0.09, by intention-to-treat analysis, Figure 2).

#### 3.4.3. FD-QoL Scores

After 6 weeks of treatment, the FD-QoL scores in both groups had improved (27.74 ± 18.63 at 0 week to 18.91 ± 17.58 at 6 weeks in BST group versus 32.18 ± 17.31 at 0 week to 18.51 ± 14.68 at 6 weeks in the placebo group); no significant difference was observed between the 2 groups (*P* = 0.89, by intention-to-treat analysis, Figure 2).

#### 3.4.4. Changes in GMA

The quality of the EGG recordings was high, and no high levels of motion artifacts were observed. The mean value of the DF was 3.03 ± 0.24 cpm among all of the subjects (3.04 ± 0.25 in the BST group versus 3.02 ± 0.23 in the placebo group), which is considered to be within the normal range. The percentages of normal gastric slow waves or gastric dysrhythmias for each channel in both groups are described in detail in Table 4.

In this study (*n* = 100), some dyspeptic symptoms were significantly correlated with EGG parameters (preprandial DF and bloating, *r* = 0.262, *P* = 0.017; preprandial percentage of tachygastria in channel 3 and abdominal cramps, *r* = 0.206, *P* = 0.040; preprandial percentage of arrhythmia in channel 3 and vomiting, *r* = 0.258, *P* = 0.010; postprandial percentage of tachygastria in channel 1 and sickness, *r* = 0.288, *P* = 0.004; postprandial percentage of arrhythmia in channel 3 and vomiting, *r* = 0.231, *P* = 0.021; postprandial percentage distribution of tachygastria in channel 3 and early satiety, *r* = 0.241, *P* = 0.016, Pearson's correlation coefficient).

There was a pattern of decreasing tachygastria in the BST group compared with the placebo group at 6 weeks (preprandial tachygastria in channel 3, *P* = 0.068; postprandial tachygastria in channel 2, *P* = 0.065; postprandial tachygastria in channel 3, *P* = 0.041). No significant differences among the pre- and postprandial EGG recordings were observed between the 2 groups at 6 weeks. 

Abnormal EGG findings were defined as a reading of dysrhythmia in ≥30% (normogastria <70%) of the total readings in the fed state or a power ratio of less than 1 [22].

Considering the definition of abnormal EGG findings, at 6 weeks, the power ratios in channels 1 and 2 for the BST group tended to be improved, whereas those for the placebo group were aggravated (Table 4).

## 4. Discussion

This study demonstrated that BST administration for 6 weeks improved dyspeptic symptoms and the quality of life for FD patients. However, verification of the significant effects of BST on the overall FD symptoms and quality of life compared with placebo was difficult, because the placebo response rate was relatively high in this trial. Nonetheless, in the BST group, early satiety was significantly ameliorated at 6 weeks, and there were significant positive changes in the GMA parameters. 

In traditional Korean medicine, BST is a well-known treatment for “epigastric stuffiness (*心下痞*),” as described in the ancient herbal prescription literature [10]. Epigastric stuffiness appears to be similar to the early satiety and abdominal discomfort symptoms reported among the symptoms of dyspepsia. Delayed gastric emptying or antral dysmotility is considered to be the main cause of this dyspepsia symptom in FD patients [19, 22].

Previous studies have indicated that BST increases levels of some gut hormones, such as motilin, gastrin, and somatostatin, which are closely correlated with gastric motility [11]. In addition, BST may regulate the hypothalamic-pituitary-adrenal (HPA) axis by increasing levels of calcitonin gene-related peptide and substance P or decreasing levels of adrenocorticotropic hormone and cortisol [12, 13]. Pinelliae tuber is a major component among the herbal components of BST and has been reported to accelerate gastrointestinal motility and gastric emptying in human studies [11, 12]. Therefore, BST or its major component, pinelliae tuber, may improve the impaired gastric motility (delayed gastric emptying or antral dysmotility) in FD patients by changing the levels of gut hormones or regulating the HPA axis, thereby alleviating the early satiety symptom in this trial.

Although the pathogenic factors of FD remain unclear, up to 50% of FD patients have gastric motility dysfunction [23]. Gastric motility dysfunction, including gastric hypomotility or uncoordinated antral-duodenal contractions, may lead to delayed gastric emptying or impaired accommodation reflexes in the proximal stomach [24]. Gastric hypomotility and uncoordinated antral-duodenal contractions in FD patients are closely associated with gastric myoelectrical dysrhythmias. These dysrhythmias arise from dysregulation of gastric slow waves, which normally occur at a frequency of 3 cpm [19]. Therefore, GMA of the FD patients in this study was measured to investigate the correlation between dyspeptic symptoms and GMA variability and to elucidate the impacts of BST on gastric slow wave before and after administration of BST or placebo.

EGG is a diagnostic technique that can record GMA obtained from cutaneous abdominal electrodes [22]. Although some controversy exists regarding the correlation between GMA obtained from cutaneous EGG and dyspeptic symptoms [25], other clinical researchers have demonstrated a positive correlation between the frequency information obtained from cutaneous EGG and myoelectrical signals acquired directly from gastric serosal leads [26, 27].

During the fasting or fed states, the normal stomach muscles emit regular gastric slow waves and spike potentials and then periodically contract like heart muscles [22]. Multichannel, cutaneous EGG is a useful and noninvasive diagnostic technique that records GMA, consisting of gastric slow waves and spikes, through the abdominal surface [22]. In healthy subjects, a normal GMA is defined as approximately 2–4 cpm of the dominant frequency or >70% of normal gastric slow waves in a total EGG recording or a power ratio of >1 (defined as the ratio of the postprandial to fasting power of the DF) [22]. However, in FD patients, abnormal EGG findings, including excessive gastric dysrhythmia (especially tachygastria) or lowered postprandial dominant power, have been reported in many studies. These abnormal findings are closely correlated with delayed gastric emptying, impaired gastric accommodation, or antral hypomotility [19, 22, 28]. In our study, the DFs of both groups were within the normal range, whereas abnormal findings were observed in the pre/postprandial gastric slow waves (gastric dysrhythmia) and power ratios. Gastric dysrhythmia in channel 3 was one of the most common findings in this study. These results were quite similar to those of many previous EGG studies examining FD or gastric motility disorders [19, 22]. Moreover, the percentage of the normal power ratio was relatively low in the BST group compared with the placebo group; this may be due to a significantly high body mass index in the BST group. This observation was similar to those in a previous study examining the relationship between EGG power ratio and BMI (Table 1) [29]. After a 6-week administration of BST, early satiety was significantly ameliorated, the percentage of gastric dysrhythmia (including tachygastria) decreased, and the abnormal power ratios were improved (Tables 2 and 4). Early satiety symptoms are related to impaired gastric accommodation or delayed gastric emptying following a meal [19, 22]. Under these pathogenic conditions in FD patients, tachygastria or a lowered power ratio (<1) in the EGG findings has been frequently observed in many studies [19, 22]. In addition, EGG channel 3 is considered to be approximately located over the surface of the distal antrum. The distal antrum contains an ectopic pacemaker that generates the gastric slow waves in the stomach [19]. Therefore, BST may be postulated to modulate the irregularities in the ectopic pacemaker in the gastric antrum and decrease gastric dysrhythmia (especially tachygastria) or increase the power ratio and then improve dyspeptic symptoms such as early satiety.

In contrast, there were no significant differences in EGG findings according to the FD subtypes (data not shown). PDS or mixed FD subtypes were dominant in both groups (76% in the BST group and 84% in the placebo group), and these FD subtypes may be correlated to gastric motility dysfunction. Alternately, each subtype group may not have represented a large enough sample size to allow determination of statistically meaningful results. Therefore, a larger sample size for comparison of EGG parameters in other FD subtypes, such as EPS, is required in future studies. Our results suggest that EGG can be used for the assessment of interventional efficacy by detection of abnormal gastric slow waves.

In our study, there was a high placebo response rate with regard to the improvement in FD symptoms and QoL. In general, a 30–40% placebo response rate has been reported in many FD clinical trials [21]. The possible factors contributing to a high placebo response include natural history, Pavlovian conditioning, regression to the mean, small sample sizes, high expectations, longer administration durations, and a high number of visits and augmented relationships with doctors [30, 31]. As shown in Table 3, a relatively high percentage of subjects in the placebo group thought that they had received BST in our study. Additionally, other factors, such as an augmented relationship with doctors, may lead to a higher placebo response rate. Therefore, recruitment of subjects not exposed to experimental interventions, or clinical trial designs that control the relationship between the doctor and the patients will be needed in future studies.

Our study may be contrasted to a previous report regarding BST (*Ban xia xie xin* decoction in traditional Chinese medicine) [14]. First, we did not show a significantly beneficial effect of BST on FD patients compared with placebo; significant improvements in overall symptoms of FD were reported in the earlier study. This may be due to differences in the components of the herbal preparation used in the previous study. Although the name of the herbal preparation used in both studies was the same, the components were different. Furthermore, the use of 3 additional herbs (cortex *Magnolia officinalis*, medicated leaven, and ark shell) in the previous study may have positively or synergistically caused the improvement in FD symptoms; these herbs have been reported to have beneficial effects on functional gastrointestinal disorders [8]. Second, there was an obvious difference in the study populations between the 2 studies. Our study was aimed towards all FD patients, whereas the previous study targeted a specific group of FD patients, based on traditional Chinese medicine classifications. In addition, the duration of symptoms in our study (13.82 ± 3.25 years in the BST group) was much longer than that in the previous study (46.67 ± 59.41 months in the modified BST group). This indicated that the FD patients in our study had more severe dyspepsia symptoms, possibly affecting the efficacy of BST in our study. Third, there were markedly different placebo response rates in the 2 studies. Our study showed a high placebo response rate (about 50% improvement), whereas only a 30% improvement in the placebo group was observed in the previous study. As mentioned above, other factors may have caused the high placebo response rate observed in our study. The high placebo response rate caused the BST to be statistically ineffective for the treatment of FD, despite similar improvements in FD symptoms compared with the previous study. Fourth, a specific diagnostic assessment, namely, GMA measurements by EGG, was conducted for FD patients in our study. There are several causes of FD development; however, gastric motility dysfunction, including delayed gastric emptying, antral dysmotility, or impaired meal accommodation, is the major cause of FD in Asians [28]. Therefore, the findings of our study will be useful for treating FD from the viewpoint of gastric motility dysfunction.

## 5. Conclusions

Compared with placebo, BST did not show a significant effect on FD. However, early satiety in FD patients may have improved after BST administration. In addition, EGG may be a useful modality for assessing the effects of therapeutics on gastric motility dysfunction. Further investigation focused on specific dyspeptic symptoms and related gut hormones, larger scale studies of FD subtypes, or development of the Zheng subgroup scale are required.

## Figures and Tables

**Table 1:** Characteristics of the subjects.

Parameters	BST (*n* = 50)	Placebo (*n* = 50)	*P* value
Gender (male/female)	25/25	22/28	0.55
Age (years)	49.54 ± 14.72	48.00 ± 12.62	0.53
Weight (kg)	61.88 ± 11.56	57.35 ± 9.23	0.03*
Height (cm)	163.67 ± 7.32	163.92 ± 7.73	0.86
Heart rate/min	73.76 ± 11.63	75.02 ± 11.42	0.70
BP systolic (mmHg)	123.44 ± 5.08	118.27 ± 11.67	0.06
BP diastolic (mmHg)	78.94 ± 13.36	74.12 ± 11.23	0.08
Smoking (yes/no)	7/43	6/44	0.77
Alcohol (yes/no)	25/25	24/26	0.84
Coffee (yes/no)	34/16	33/17	0.83
Rome criteria			
Postprandial distress syndrome (PDS) (%)	21 (42.00)	27 (54.00)	0.43
Epigastric pain syndrome (EPS) (%)	12 (24.00)	8 (16.00)	
Mixed (PDS + EPS) (%)	17 (34.00)	15 (30.00)	
Duration of symptom (years)	13.82 ± 3.25	13.37 ± 13.68	0.88
Characteristics of dyspepsia (%)			
Continuous	21 (40.91)	17 (32.50)	0.10
Periodic	16 (34.09)	10 (20.00)	
Irregular	13 (25.00)	23 (47.50)	
*Helicobacter pylori* infection			
In history (%)	8 (16.00)	11 (22.00)	0.74
Total GIS score	13.44 ± 4.87	13.82 ± 5.05	0.69
VAS for overall symptom	55.36 ± 18.63	53.08 ± 17.29	0.38
Total FD-QoL score	27.74 ± 18.63	32.18 ± 17.31	0.54
Total BDI score	22.70 ± 12.82	25.04 ± 13.49	0.30

BST: *Banha-sasim-tang*; BP: blood pressure; GIS: gastrointestinal symptom scale; VAS: visual analogue scale; FD-QoL: functional dyspepsia-related quality of life; BDI: Beck's depression inventory.

*Statistically significant.

**Table 2:** Changes in individual dyspeptic symptoms of gastrointestinal symptom (GIS) scale.

Symptoms	Baseline		6 weeks		14 weeks	
	BST (*n* = 50)	Placebo (*n* = 50)	BST (*n* = 44)	Placebo (*n* = 41)	BST (*n* = 44)	Placebo (*n* = 40)
Nausea	1.04 ± 1.10	1.32 ± 1.02	0.70 ± 0.93	0.59 ± 0.84	0.59 ± 0.84	0.60 ± 0.71
Sickness	0.60 ± 0.90	0.62 ± 0.88	0.50 ± 0.82	0.49 ± 0.81	0.48 ± 0.73	0.30 ± 0.65
Vomiting	0.56 ± 0.86	2.38 ± 1.27	0.36 ± 0.72	0.22 ± 0.52	0.25 ± 0.53	0.15 ± 0.43
Bloating	2.42 ± 0.93	2.38 ± 1.03	1.43 ± 0.97	1.15 ± 0.96	1.57 ± 1.11	1.20 ± 0.88
Abdominal cramps	1.02 ± 0.94	1.32 ± 1.30	0.66 ± 0.94	0.54 ± 0.87	0.48 ± 0.59	0.45 ± 0.78
Early satiety	2.30 ± 1.28	2.26 ± 0.92	**1.09 **± 0.86*	1.59 ± 0.84	1.16 ± 1.14	1.28 ± 0.96
Heart burn	1.50 ± 1.16	1.50 ± 1.28	0.89 ± 0.89	0.71 ± 0.87	0.77 ± 0.94	0.78 ± 0.97
Loss of appetite	1.40 ± 1.21	1.34 ± 1.21	0.91 ± 1.01	0.71 ± 0.90	0.77 ± 0.94	0.83 ± 0.96
Retrosternal discomfort	1.00 ± 1.14	0.84 ± 1.17	0.66 ± 1.03	0.39 ± 0.67	0.59 ± 0.84	0.38 ± 0.63
Epigastric or upper abdominal pain	1.60 ± 1.01	1.68 ± 1.33	0.98 ± 1.07	0.71 ± 0.87	0.66 ± 0.96	0.53 ± 0.75

BST: *Banha-sasim-tang*.

*Statistically significant.

**Table 3:** Comparison between actual administration of experimental agents and subjects' expectation about their own groups at the end of the study.

		Subjects' expectation			*P* value
		BST *n*, (%)	Placebo *n*, (%)	Total *n*, (%)	
Actual administration	BST *n*, (%)	24 (28.6)	20 (23.8)	44 (52.4)	0.15
	Placebo *n*, (%)	28 (33.3)	12 (14.3)	40 (47.6)	

	Total *n*, (%)	52 (61.9)	32 (38.1)	84 (100)	

BST: *Banha-sasim-tang*.

**Table 4:** Comparison of main parameters in electrogastrography (EGG) between BST and placebo groups.

EGG parameters	Groups	Normal/abnormal	Electrode sites of EGG					
			Channel 1		Channel 2		Channel 3	
			0 week	6 weeks	0 week	6 weeks	0 week	6 weeks
Preprandial dominant frequency *n*, (%)	BST	Normogastria	37 (74.0)	30 (69.8)	29 (58.0)	25 (56.8)	29 (58.0)	25 (56.8)
		Dysrhythmia	13 (26.0)	13 (30.2)	21 (42.0)	19 (43.2)	21 (42.0)	19 (43.2)
	Placebo	Normogastria	40 (80.0)	31 (75.6)	41 (82.0)	28 (68.3)	34 (68.0)	31 (75.6)
		Dysrhythmia	10 (20.0)	10 (24.4)	9 (18.0)	13 (31.7)	16 (32.0)	10 (24.4)

Postprandial dominant frequency *n*, (%)	BST	Normogastria	35 (70.0)	35 (79.5)	35 (70.0)	32 (72.7)	33 (66.0)	36 (81.8)
		Dysrhythmia	15 (30.0)	9 (20.5)	15 (30.0)	12 (27.3)	17 (34.0)	8 (18.2)
	Placebo	Normogastria	38 (76.0)	31 (75.6)	34 (68.0)	30 (73.2)	40 (80.0)	34 (82.9)
		Dysrhythmia	12 (24.0)	10 (24.4)	16 (32.0)	11 (26.8)	10 (20.0)	7 (17.1)

Power ratio *n*, (%)	BST	≥1	25 (59.5)	24 (74.3)	22 (57.9)	22 (67.6)	24 (75.0)	24 (67.6)
		<1	17 (40.5)	11 (25.7)	16 (42.1)	12 (32.4)	12 (25.0)	13 (32.4)
	Placebo	≥1	31 (72.7)	22 (59.5)	30 (73.2)	17 (50.0)	33 (85.0)	24 (72.2)
		<1	13 (27.3)	15 (40.5)	11 (26.8)	19 (50.0)	7 (15.0)	12 (27.8)

Abnormal EGG findings are defined as a reading of gastric dysrhythmia (including tachygastria, bradygastria, and arrhythmia) in ≥30% (or normogastria < 70%) of the total recordings of EGG in the fed state or a power ratio of >1 [22].

BST: *Banha-sasim-tang*.
